# Insights into the biodegradation of weathered hydrocarbons in contaminated soils by bioaugmentation and nutrient stimulation

**DOI:** 10.1016/j.chemosphere.2016.07.032

**Published:** 2016-10

**Authors:** Ying Jiang, Kirsty J. Brassington, George Prpich, Graeme I. Paton, Kirk T. Semple, Simon J.T. Pollard, Frédéric Coulon

**Affiliations:** a School of Water, Energy and Environment, Cranfield University, Cranfield, MK43 0AL, UK; b Institute of Biological and Environmental Sciences, Cruickshank Building, University of Aberdeen, Aberdeen, Scotland, AB24 3UU, UK; c Lancaster Environment Centre, Lancaster University, Lancaster, LA1 4YQ, UK

**Keywords:** Soil contamination, Bioremediation, Weathered petroleum hydrocarbon, Bioaugmentation, Biostimulation

## Abstract

The potential for biotransformation of weathered hydrocarbon residues in soils collected from two commercial oil refinery sites (Soil A and B) was studied in microcosm experiments. Soil A has previously been subjected to on-site bioremediation and it was believed that no further degradation was possible while soil B has not been subjected to any treatment. A number of amendment strategies including bioaugmentation with hydrocarbon degrader, biostimulation with nutrients and soil grinding, were applied to the microcosms as putative biodegradation improvement strategies. The hydrocarbon concentrations in each amendment group were monitored throughout 112 days incubation. Microcosms treated with biostimulation (BS) and biostimulation/bioaugmentation (BS + BA) showed the most significant reductions in the aliphatic and aromatic hydrocarbon fractions. However, soil grinding was shown to reduce the effectiveness of a nutrient treatment on the extent of biotransformation by up to 25% and 20% for the aliphatic and aromatic hydrocarbon fractions, respectively. This is likely due to the disruption to the indigenous microbial community in the soil caused by grinding. Further, ecotoxicological responses (mustard seed germination and Microtox assays) showed that a reduction of total petroleum hydrocarbon (TPH) concentration in soil was not directly correlable to reduction in toxicity; thus monitoring TPH alone is not sufficient for assessing the environmental risk of a contaminated site after remediation.

## Highlights

• Improved biodegradation of weathered hydrocarbon residues.
• Nutrient addition is a key parameter for promoting biodegradation.
• Soil grinding reduced effectiveness of biostimulation.
• Reduction of petroleum hydrocarbons in soil not directly correlable to reduction in toxicity.

## 1 Introduction

Land contamination from poor historical industrial practices or incidents is a widespread and well recognised environmental issue. In the UK alone, it has been estimated that ca. 300,000 ha of land could be affected by industrial activity leading to contamination (Environment Agency, 2009). Petroleum hydrocarbons are one the most common contaminant, though a wide range of chemicals may be present (Towell et al., 2011). Once released into the environment, petroleum hydrocarbons are subject to abiotic and biotic weathering reactions e.g. physical and biochemical transformations, interactions with soils, that will change their composition and toxicity, and will influence their fate and biodegradation (Brassington et al., 2007, Stroud et al., 2007, Maletić et al., 2011). The extent of these transformations will vary according to the type of petroleum products present, the soil conditions (e.g. organic matter content, soil grain size and clay-type at the sites) (Stroud et al., 2007), and the bioavailability and susceptibility of the different compounds (Stroud et al., 2007, Maletić et al., 2011).

Bioremediation has become the preferred method for the remediation of petroleum hydrocarbon contaminated soils, because it is considered cost effective and sustainable, and can accelerate naturally occurring biodegradation processes through the optimisation of limiting parameters (Vidali, 2001, Coulon et al., 2012). To be effective, it is important to investigate and understand all factors (e.g. soil and contaminant characteristics, bioavailability, stage of weathering) that might effect the efficacy of the process. For example, aliphatic hydrocarbons of intermediate length (ranging between C_10_ and C_25_) tend to be readily degradable by microorganisms despite their low solubility, whereas longer chain alkanes (C_25_-C_40_), especially those with branched or cyclic chain structures, are more resistant to biological degradation (Maletić et al., 2011).

Heavily weathered hydrocarbons are difficult to biodegrade and have relatively low toxicity, but high residual concentrations can severely alter the physical and chemical properties of the soils, thus reducing soil fertility (Coulon et al., 2012, Wu et al., 2013). Remediation outcomes using biological methods for the treatment of weathered hydrocarbons are often unpredictable, and in some instances contaminated soil may be regarded as ‘untreatable’ via bioremediation (Brassington et al., 2007). Debate around the benefits of bioaugmentation and its capacity to increase the microbial degradation of weathered hydrocarbons after indigenous microorganisms are no longer effective continues, and only a few studies demonstrate continued biodegradation after introduction of specific hydrocarbon degraders (Coulon et al., 2012, Gallego et al., 2001). Biodegradative performance via bioaugmentation can be further improved by the addition of appropriate nutrients; a process referred to as biostimulation (Xu and Lu, 2010). Due to the limited number of studies on the subject, and the complexity of weathered petroleum hydrocarbon products, there is a need for investigation into the potential for bioaugmentation coupled with biostimulation to enhance biotransformation and reduce residual toxicity.

In this study, we investigated the potential for biotransformation of weathered hydrocarbon residues in soil. To do so, we determined whether it was possible to improve biodegradation with the simultaneous application of bioaugmentation and biostimulation on two soil types. Soil A was taken from a windrow where bioremediation had been completed and soil B was taken from a site prior remediation where oil drums had leaked contaminating the soil. Soil A treatment was deemed completed as no further degradation could be achieved. This research provides valuable knowledge concerning chemical and toxicological changes on a soil type not previously investigated and could be used to support the development of bioremediation strategies. Finally, we discuss the relationship between chemical change, toxicity, and total petroleum hydrocarbons (TPH) measurements in the context of risk assessment, highlighting the effects that remediation might have on soil toxicity.

## 2 Materials and methods

### 2.1 Physical and chemical characterisation of soil samples

Two different soils collected at a depth of 5–20 cm from two commercial oil refinery sites located in the UK were labelled A and B (to maintain owner anonymity). Soil A is a sandy soil which was heavily contaminated with weathered hydrocarbons (TPH = 50,000 mg kg^−1^). After 6 month windrow treatment, TPH concentration was decreased to 22,700 mg kg^−1^ where it was believed no further degradation was possible. Soil B is predominantly clay soil contaminated with weathered hydrocarbons (TPH = 31,500 mg kg^−1^) taken from a more recently contaminated site where there was no history of any remedial activity. The soils were air-dried for 24 h and sieved through 2 mm mesh to remove stones, plant material, and to facilitate mixing. Prior to air drying the field moisture content was determined in triplicate by oven drying at 105 °C for 24 h. Soils were then stored at 4 °C in the dark before use.

For both soil samples, a routine set of characterisation was carried out. Soil pH was measured using a pH meter (Jenway 3540) in a distilled water slurry (one part soil: two parts water) after a 30 min equilibration period (Black, 1965). Maximum water holding capacity (WHC) was determined in duplicate by flooding the wet weight equivalent of 100 g of dry soil in a filter funnel and allowing it to drain overnight (Black, 1965). Particle size analysis was performed by a combination of wet sieving (sand) and sedimentation (silt and clay), as described by Gee and Baude (1986). The organic matter content as indicated by loss on ignition (LOI) of each soil was measured by combustion at 450 °C in a furnace for 24 h, according to ASTM Method D297487. Total organic carbon (TOC) was analysed by potassium dichromate oxidation, as described by Schnitzer (1982).

For nitrate, phosphate, and ammonium determination, 10 g of soil was first extracted in 0.5 M potassium bicarbonate (adjusted to pH 8.5). The extractant was then analysed by high-performance liquid chromatography (HPLC) for nitrate and phosphate as described by Brenner and Mulvaney (1982) and Olsen and Sommers (1982), respectively. Ammonium was analysed using the colorimetric test described by Reardon et al. (1966).

### 2.2 Microcosm experiment design

Soil microcosms were established using 700 g of either soil A and B in sterile 1 L, wide-mouth amber glass jars. Four different microcosm conditions for each soil were established and tested in triplicate (Table 1). Soil grinding was done using mortars and pestles made from hard chemical-porcelain ware. The mortars had a lip and were glazed on the outside. The pestles were glazed to the grinding surface. Soil aliquots of about 70 g were ground for about 15 min in the mortar with the pestle to pass the soil through a 42.5 μm sieve. The ground soil aliquots were then combined for additional sample preparation. Nutrients were added in the form of ammonium nitrate and potassium orthophosphate to obtain a C:N:P ratio of 100:1:0.1. The hydrocarbon-degrading inoculum was composed of three bacterial isolates supplied by Remedios Limited (Aberdeen). The bacterial isolates were isolated from an attenuated enrichment culture from No.6 oil impacted soil (Alzahrany, 2011). Two bacterial isolates were related to Pseudomonas sp. (100% match) and one to Klebsiella sp. (99% match). Inoculum was grown using a minimal medium supplemented with diesel (equivalent to 5.5 mg C l^−1^) as carbon source. The cell concentration added to each microcosm was such as to give 5 × 10^7^ CFU g^−1^ soil. For each amendment, a few woodchips were added to 10 ml of Bushnell-Haas broth supplemented with 1 g l^−1^ salicylic acid and 1% ethanol, adjusted to pH 7. The mixture was placed in an orbital shaker at 150 rpm in the dark at 20 °C and left overnight, after which 1 ml was added to 100 ml of fresh medium and grown to a stationary phase (about 24 h, checked by optical density readings at 600 nm). The cell number at stationary phase was 10^8^ cells ml^−1^. The inoculum solution was then added to the soils at 0.01 ml g^−1^ dry wt soil to achieve 10^6^ cells g^−1^ dry wt soil. The moisture content of each microcosm was adjusted to 80% of the soil’s water holding capacity using deionised water. The microcosms were incubated in the dark at 15 °C. High humidity was maintained using damp cotton wool and moisture checked periodically. Each microcosm was mixed weekly and capped loosely to allow oxygen transfer. Soil from each microcosm was sampled at 0, 7, 14, 28, 56, and 112 days for subsequent microorganism respiration monitoring and hydrocarbon analysis.

### 2.3 Microbial respiration monitoring

A soil sample (1 g on dry weight basis) from each microcosm was collected and sealed in a headspace vial. All vials containing microcosm samples were incubated under the same conditions as the microcosms for 24 h. For CO_2_ analysis, the headspace gas was sampled with a gastight syringe and manually injected into a Cambridge Scientific 200 series gas chromatograph with thermal conductivity detector (GC-TCD; Cambridge Scientific Instruments, Cambridge, UK) using helium as carrier gas at 20 psi (138 kPa). The GC was fitted with a CTR1 concentric packed column (Alltech, USA). The column oven and injector temperature were 110 °C and 125 °C, respectively. The GC was calibrated using a standard CO_2_ (1% CO_2_ balanced with N_2_). Respiration values were determined on a mg CO_2_ kg soil^−1^ day^−1^ basis following subtraction of a blank vial containing atmospheric CO_2_ only.

### 2.4 Microbial enumeration

Homogenized soil (2 g) was weighed into a glass Universal bottle and 20 ml of ¼ strength Ringer’s solution was added. Samples were then vortexed for 30 s and sonicated for 1 min and allowed to stand for a further 2 min. A 100 ml aliquot of soil suspension was removed and serially diluted in ¼ strength Ringer’s solution to the appropriate dilution factor (10^−5^ or 10^−4^ dilution factor). An aliquot of 10 ml of each dilution series was added in triplicate to ¼ strength Luria Bertani medium to determine heterotrophs and Bushnell-Hass with 1% diesel as the sole carbon source for hydrocarbon-degraders. Samples were incubated at 25 °C for 24–48 h thereafter and colony-forming units (CFUs) enumerated. Results are expressed as CFU g^−1^ of dry soil.

### 2.5 Hydrocarbon analysis

Hydrocarbon extraction was performed as described by Risdon et al. (2008). Briefly soils (5 g) were chemically dried with 5 g anhydrous Na_2_SO_4_ in 50 ml Teflon centrifuge tubes. Acetone (4 ml) was added and sonicated for 2 min at 20 °C. Acetone (6 ml) and hexane (10 mL) were added to the samples and sonicated for 10 min, followed by manually shaking to mix the solvent and soil. This step was repeated twice followed by centrifugation for 5 min at 750 rpm. After passing the supernatant through a filter column fitted with glass receiver tube, a sequential step series, including resuspension of the samples in 10 ml of acetone/hexane (1:1), sonicated for 15 min at 20 °C, centrifugation for 5 min at 750 rpm, and decantation into a filter column, was repeated twice. The final extract volume was adjusted to 40 ml with a mixture of acetone/hexane (1:1) and homogenized by manual shaking. The silica gel column clean-up was performed by passing the extracts through a column filled with florisil. Total extractable and recoverable petroleum hydrocarbons (TERPH), aliphatic and aromatic fractions were identified and quantified using a Perkin Elmer AutoSystem XL gas chromatograph coupled with a Perkin Elmer Turbomass Gold mass spectrometer operated at 70 eV in positive ion mode. The GC was fitted with a Restek RTX -5MS capillary column (30 m in length, 0.25 mm internal diameter and 0.25 μm coating). Splitless injection with a sample volume of 1 μl was applied. The oven temperature was increased from 60 °C to 220 °C at 20 °C min^−1^, then to 310 °C at 6 °C/min and held at this temperature for 15 min. The mass spectrometer was operated using the full scan mode (range *m/z* 50–500) for quantitative analysis of target alkanes and PAHs. For each compound, quantification was performed by integrating the peak at specific *m/z*. External multilevel calibrations were carried out for both oil fractions, quantification ranging from 0.5 to 2500 μg ml^−1^ and from 1 to 5 μg ml^−1^, respectively. Internal standards for the alkanes were nonadecane-d_40_, triacontane-d_62_ and naphthalene d_8_, phenanthracene-d_10_, chrysene-d_12_ and perylene d_12_ (Sigma Aldrich, Dorset, UK). For quality control, a 500 μg ml^−1^ diesel standard and mineral oil were analysed every 20 samples. In addition, duplicate blank controls were also performed by going through the same extraction procedure but containing no soil. The reagent control was treated following the same procedure as the samples without adding soil sample. The reference material was an uncontaminated soil of known characteristics, and was spiked with a diesel and mineral oil standard at a concentration equivalent to 16,000 mg kg^−1^.

### 2.6 Soil toxicity bioassay

Seed germination and Microtox^®^ assay were carried out at the start and the end of the microcosm experiment. The selection of the ecotoxicity assays were based on their ease of execution and representation of different ecological soil organisms. Seed germination tests were performed according to Saterbak et al. (2009). Ten white mustard seeds (*Brassica alba*) were planted into 20 g of test soil (wet weight) in 120 ml bottle. This was repeated 10 times. The seeds were left to germinate for 4 days at 25 °C in darkness. At the end of each test, if a root was visible, the seed was scored as germinated. Microtox^®^ solid phase test (Microtox^®^ SPT) assay was carried out according to Azur Environmental (1998). Tests were done in triplicate. The soil dilution that inhibits 50% (EC_50_) of the light output relative to oil-free soil collected nearby the sampling sites was calculated for each oiled samples and expressed as a percent of the pristine sample. Note that Microtox EC_50_ values decline as toxicity increases. A standard 100 g l^−1^ phenol solution was used to check the performance of both operator and analytical system and the 95% confidence range was maintained below 15% variation throughout the study.

### 2.7 Statistical analysis

Statistical analysis of the results such as mean, standard deviation (SD), standard error (SE) and analysis of variance (ANOVA) were performed using Excel and SPSS (version 17, Statistical Product and Service Solutions). Differences in the TERPH, alkanes and PAH concentration between different treatments were compared using ANOVA by Fishers Least Significant Difference (LSD) test. The difference was recognised as significant where P < 0.05.

## 3 Results and discussion

### 3.1 Soil characteristics

**Fig. 1:**
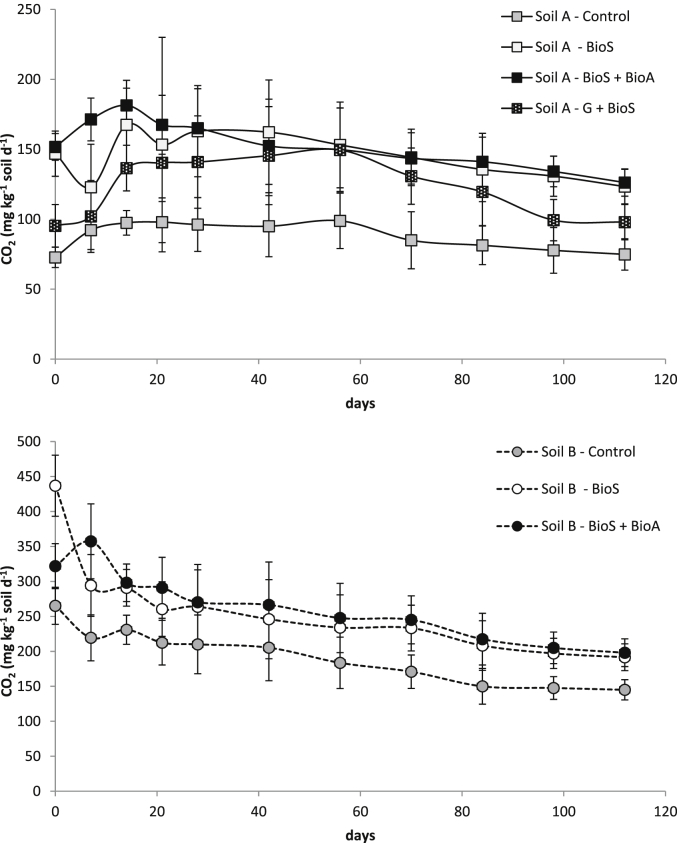
Mean CO_2_ release over 112 days (Error bars indicate the standard deviation of triplicate measurements).

Soil characterisation provided baseline physical and chemical properties of the two soil samples used in this study (Table 2). TPH concentration in Soil A was measured at 22,700 mg kg^−1^, and for Soil B, at 31,500 mg kg^−1^. Both values indicate that the two soils contained elevated concentrations of petroleum hydrocarbons (Zemanek et al., 1997, Tindal, 2005). The ammonium and nitrate levels were undetectable in both soils, phosphate was undetectable in Soil B and measured at a low level (0.0016 mg kg^−1^) in Soil A. These conditions suggest that both soils could benefit from biostimulation with nutrients. Biodegradation within each soil was not seen to be limited by carbon, nitrogen, pH or moisture conditions as these remained within acceptable ranges (Eweis et al., 1998, Coulon et al., 2012, Wu et al., 2013). Microbial respiration tests (Table 2 and Fig. 1) indicated that an active microbial population was present within both soils prior to the addition of microbial inoculum (Diploick et al., 2009). Other soil properties are shown in Table 2. More detailed soil analysis results including metal concentrations have been reported in a previous study (Towell et al., 2011).

### 3.2 Soil microcosm experiment

#### 3.2.1 Microbial respiration and counts

All of the microcosms contained a viable microbial community as demonstrated by respiration rates that were measured by CO_2_ production from the soil samples (Table 2 and Fig. 1). Soil A (<200 mg CO_2_ kg soil d^−1^) had a lower respiration rate than Soil B (∼275 mg CO_2_ kg soil d^−1^) and this was likely due to the higher concentration of longer chain hydrocarbons (Fig. 3) and other recalcitrant fractions, as a result of a longer exposure to contamination and weathering. In contrast, Soil B was contaminated more recently and the presence of short chain hydrocarbons and other readily biodegradable compounds (Fig. 3) could be responsible for the initially higher level of CO_2_ production (Fig. 1). Analysis of the initial soil hydrocarbon concentrations and hydrocarbon fractions support this claim (Table 3).

**Fig. 2:**
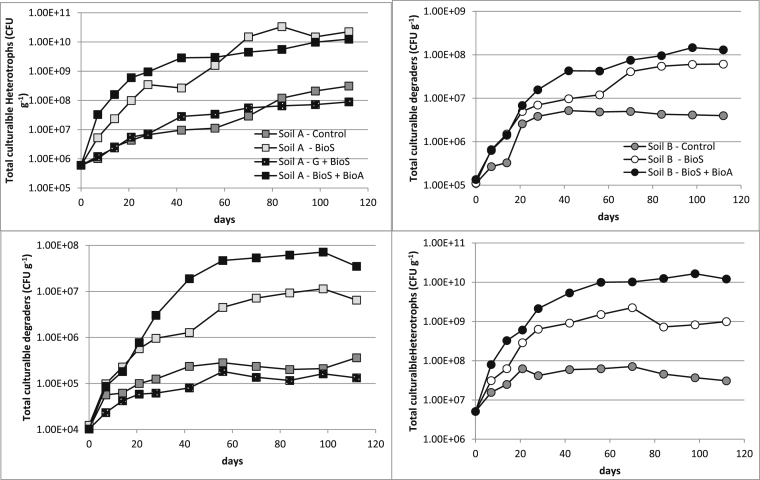
Total culturable heterotroph and degrader numbers over 112 days (Standard deviation was <20% for both CFU counts and not shown on the graphs for clarity).

The amendment strategies applied to both soils, except soil grinding + biostimulation, increased by two and three orders of magnitude the numbers of culturable heterotrophs and hydrocarbon degraders (Fig. 2) and this translated into enhanced CO_2_ production (up to 40% increase after 14 days in Soil A microcosms and up to 23% increase after 14 days in Soil B microcosms) when compared to natural attenuation processes alone (Fig. 1). A significant increase in hydrocarbon degraders was observed within 42 days in both soils (Fig. 2). At the end of experiment, the hydrocarbon degraders number in both soils were three orders of magnitude higher than those in control soils (natural attenuation) and in soil where grinding and biostimulation was applied (10^5^–10^6^ versus 10^8^ CFU g^−1^ soil). In addition, CO_2_ production in Soil B stabilised at ∼250 mg CO_2_ kg soil d^−1^ for 56 days and then after 100 days gradually stabilised at ∼200 mg CO_2_ kg soil d^−1^ (Fig. 1). A similar trend was observed in Soil A where CO_2_ production stabilised ∼150 mg CO_2_ kg soil d^−1^ after 28 days. Where grinding and biostimulation were applied (G + BioS) to Soil A, respiration levels stabilised at ∼120 mg CO_2_ kg soil d^−1^ and after 98 days was closer to 100 mg CO_2_ kg soil d^−1^ in line with respiration levels measured with the microcosms where natural attenuation was applied (Fig. 1). The TPH levels for Soil A microcosms were similar across the four studied conditions. Thus, this suggests that grinding changed the soil and hydrocarbons intrinsic physico-chemistry that effected both the microbial activity (Fig. 1) and the microbial abundance (Fig. 2).

#### 3.2.2 Petroleum hydrocarbon analysis

**Fig. 3:**
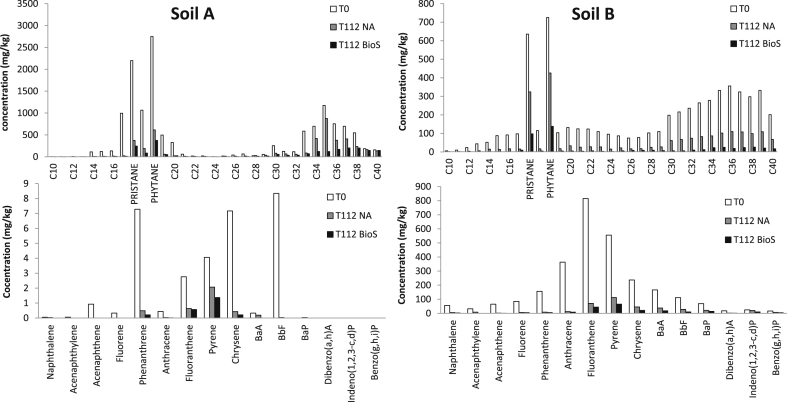
n-alkanes and PAH fingerprints in soil A and B respectively after 112 days of natural attenuation (T112 NA) and biostimulation treatment (T112 BioS).

Soil B hydrocarbons distribution showed a well-developed series of n-alkanes distribution (Fig. 3). The distribution is heavy-end skewed and bi-modal with a higher proportion of C28-C40 *n*-alkanes. In contrast soil A hydrocarbons distribution confirms that the hydrocarbon source is weathered (degraded) (Brassington et al., 2010). More specifically, the concentration of aliphatic compounds in Soil A (13,800 mg kg^−1^) was 2.2 times higher than in Soil B (Table 3). In contrast the concentration of aromatic compounds in Soil B was 100 times higher than in Soil A (Table 3). After treatment, the most prominent residual hydrocarbon fractions in Soil A and Soil B were the aliphatic fractions C_16_-C_35_ and C_35_-C_40_, and the aromatic fractions C_16_-C_21_ and C_12_-C_16_, respectively (Table 3 and Fig. 2). The largest reduction in both the aliphatic and aromatic fractions were obtained in the BioS and BioS + BioA microcosms (Table 3, Table 4). While the BioS + BioA combination improved bioremediation performance (compared to natural attenuation alone) the addition of microbes did not necessarily provide additional improvement to the biodegradation process compared to the addition of nutrients alone, suggesting that nutrient addition is a key parameter for promoting biodegradation (Hejazi and Husain, 2004). Similar hydrocarbons loss percentage results were observed in Soil B (Table 3) which had not undergone remediation previously. Further to this, the percentage of degradation (Table 3) and the degradation constant (Table 4) suggests that the higher concentrations of aromatics did not limit the extent of bioremediation performance in soils.

Even though Soil A was previously remediated and the soil had undergone considerable weathering, further degradation of the residual hydrocarbons was possible when suitable conditions were provided. As a result, bioremediation end points are variable and will depend on a range of factors, most notably the nutrient levels and the availability of microbes.

**Fig. 4:**
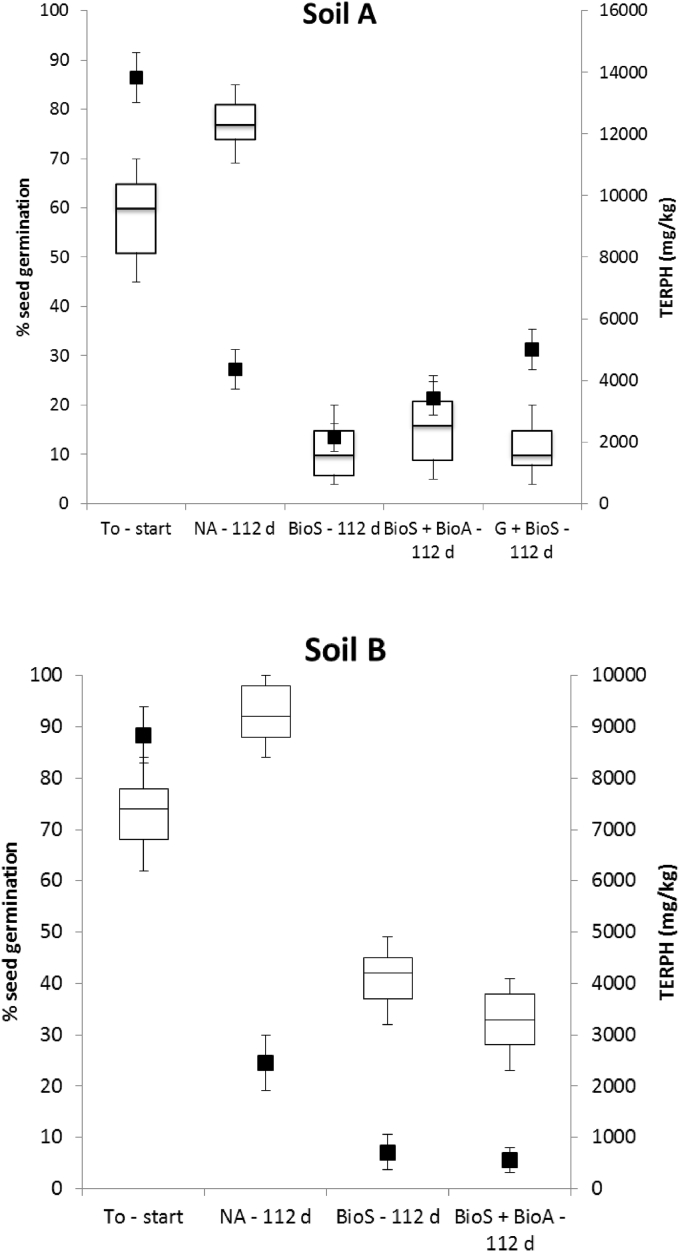
Mean seeds germination percentage (whisker boxes) and total extractable hydrocarbons resolved (aliphatic + aromatic compounds; black squares) at the onset and after 112 days for each treatment in soil A and B (error bars indicate standard error of the triplicate results).

In weathered soils, residual hydrocarbons are tightly bound to the soil matrix and can form rigid soil aggregates that can effectively entrap hydrocarbons and limit bioaccessibility (Huesemann et al., 2004). By grinding the soil, the contact surface area and oxygen transfer rates are increased and this increases the chance for microbes and hydrocarbons to come into contact (Craswell and Waring, 1972, Nasser and Mingelgrin, 2012). However, the combination of grinding and BioS was not observed to enhance the mineralisation of the aliphatic and aromatic hydrocarbon fractions compared to the natural attenuation (Table 4). This unexpected result may be due to the disruption of the indigenous microbial consortium caused by soil grinding, as previously suggested by Powlson (1980), or more likely, grinding facilitated the release of bound fractions of hydrocarbons that proved toxic to the microbes (Fig. 4a). This finding reinforces previous findings reported by Wu et al. (2013) and Huesemann et al. (2004) that state it is incorrect to assume that residual hydrocarbons after bioremediation treatment are recalcitrant and therefore they can be left in place without posing an environmental risk.

Overall, the extent of biodegradation in both soils was less significant for longer chain hydrocarbons than shorter chain hydrocarbons. This effect became more pronounced for hydrocarbon fractions with an equivalent carbon number over C_35_ as these compounds are the most recalcitrant to degradation.

#### 3.2.3 Soil ecotoxicology

It is important to evaluate soil ecotoxicology during and after any remediation treatment as it has been shown that a reduction in contaminants alone does not infer a reduction in toxicity (Coulon et al., 2005). Further to this, ecotoxicological tests can provide information on the bioavailability of contaminants present in soil (Molina-Barahona et al., 2005, Dawson et al., 2007). Results of the seed germination were normalised using a clean uncontaminated soil (control) to take into account the germination rate of the seeds used. Visual observations showed that seeds germinated quickly in the uncontaminated soil with an incidence of >90% of seed germination over the experimental period. Whilst seed germination was observed in both contaminated soils and for each treatment, it should be noted that visual observations during the course of the experiment showed that the rate of germination, and subsequently seedling growth, were reduced compared to the un-contaminated soil (control). Thus, even though Soil B, without any specific treatment (natural attenuation), achieved 100% germination at the end of the experiment (112 days) (Fig. 4), the rate and degree of growth were less than that seen in the uncontaminated soil, thus suggesting an ecotoxicological effect.

Overall, seed germination and Microtox^®^ SPT showed that the toxicity of treated soils was higher while the natural attenuation approach showed the least change in toxicity (Fig. 4). The seed germination tests showed almost 50% reduction in germination for all three treatments for Soil A and a 40% reduction in the two Soil B treatments. Therefore, although there was a considerable reduction in TERPH, the toxicity of the soils increased (Fig. 4). Such findings have been reported previously, for example, Dorn and Salanitro (2000) reported in a 360-day lab scale bioremediation trial of soils contaminated with crude oil that there was no improvement of seed germination (%) after bioremediation, although a significant degradation of contaminants had occurred. Grinding Soil A for the biostimulation condition offers one explanation for this increased toxicity. It is possible that the grinding process released toxic contaminants that were originally enclosed in soil aggregates or pores, enhancing their bioavailability.

**Fig. 5:**
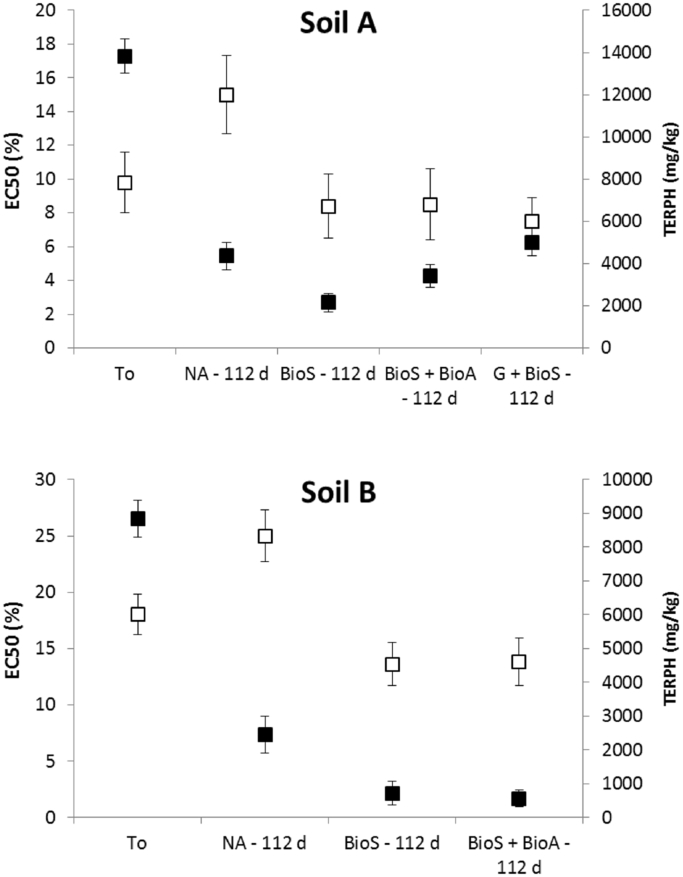
EC_50_ value (%) (white squares) and TERPH (black squares) at the onset and after 112 days for each treatment in soil A and B (error bars indicate standard error of the triplicate results; toxicity decreases when the EC_50_ value increases).

The Microtox^®^ SPT measurements are in good agreement with the findings of the seed germination in terms of ecotoxicity ranking for both soils. The EC_50_ values decreased with a decline in TERPH (Fig. 5), confirming that remediated soils have higher toxicity compared to original soil conditions and/or soil left to natural attenuation (Fig. 5). These results suggest that the negative shift is due to the compositional changes observed during the active treatments (BioS and BioS + BioA) and the by-product of biodegradation (Roy et al., 2012). In a recent study, Mamindy-Pajany et al. (2012) evaluated the ecotoxicological effects of four contaminated sediments treated with mineral additives using Microtox^®^ assay. In all treated samples, a decrease of contaminants was observed. However, the rank of toxicity was not in accordance with the rank of contamination level, and in two of the less contaminated samples an increased toxicity level was observed. Similar findings have also been reported by Xu and Lu (2010) when using Microtox^®^ SPT to evaluate the ecotoxicity of crude oil contaminated soil after bioremediation.

In sum, the results suggest that there is no direct correlation between a decrease in total extractable hydrocarbons and a reduction in toxicity. There are several causes for these discrepancies including (i) hydrocarbon bioavailability change during bioremediation treatment, (ii) complex soil-contaminant interactions as well as interactions between residual hydrocarbons rendering them more or less toxic than expected based on additive independent behaviour of toxicants, and (iii) sensitivity of the bioassay to the bioavailable fraction of the residual hydrocarbons as compared to the tightly bound fractions that could be a prominent fraction of the residual hydrocarbons (Roy et al., 2012, Mallick and Dutta, 2008, Thavamani et al., 2015). This is likely due to the more toxic intermediates formed during the biodegradation (Mallick and Dutta, 2008, Ahn et al., 2006).

## 4 Conclusion

This study confirms it is possible to improve the treatability of weathered hydrocarbons in soil by applying different bioremediation strategies such as bioaugmentation and biostimulation individually or in combination. The rates of biodegradation, however, may be affected by grinding, suggesting that the tightly bound weathered, hydrocarbon fraction can be disrupted, possibly leading to the release of toxic compounds. This observation was supported by monitoring of respiration rates and analysis of soil ecotoxicity, which confirmed that the reduction of the hydrocarbon content in soil, even for weathered hydrocarbons, does not necessarily lower the toxicity of the soil. Thus, assessing the potential biotransformation of weathered hydrocarbons in soil requires careful consideration of a wide range of factors including bioavailability change, and increased concentration of intermediates or biodegradation products during bioremediation treatment. Monitoring TPH alone is therefore not sufficient for determining the environmental risk posed by a contaminated site after remediation. Also, bioavailability is an important factor that can influence the extent of mass reduction achievable by bioremediation. However, the objective of bioremediation should not be mass reduction *per se*, but risk reduction and management. As such, it is important to consider these aspects in future research.

## Figures and Tables

**Table 1:** Microcosm experiment setup.

Condition	Soil	Amendment/Treatment detail
Natural attenuation (NA)	Soil A	None - Control
Soil B
Biostimulated (BioS)	Soil A	Amended with NH_4_NO_3_ + KH_2_PO_4_
Soil B
Biostimulated and bioaugmented (BioS + BioA)	Soil A	Same as BioS and inoculated with hydrocarbon degrader (5 × 10^7^ cells per g soil dry weight)
Soil B
Ground and Biostimulated (G + BioS)	Soil A	Same as BioS plus soil grinding

**Table 2:** Characteristics of soils A and B.

	Soil A	Soil B
TPH^a^ (mg kg^−1^)	22,700 ± 589	31,500 ± 980
Bulk density (g ml^−1^)	0.973	0.823
Moisture content in %	15 ± 2	21 ± 4
Moisture content in % at WHC^b^	38 ± 6	44 ± 8
pH in Water	6.8 ± 0.3	7.5 ± 0.2
pH in 0.01 M CaCl_2_	6.5 ± 0.0	6.6 ± 0.0
LOI in %	12 ± 0.7	15 ± 1.2
Organic Carbon in %	7 ± 2	9 ± 3
TOC^c^(μg g^−1^)	168 ± 7	280 ± 68
Carbon in %	9 ± 1	8 ± 2
Nitrogen in %	5 ± 0.5	2 ± 1
Microbial respiration (mg CO_2_ kg^−1^ soil d^−1^)	5.30	4.03
Ammonium in %	Not detected	Not detected
Nitrate in %	Not detected	Not detected
Phosphate in %	0.0016	Not detected
Total heterotrophs (CFU g^−1^)	6.0 × 10^5^	5.1 × 10^6^
Total degraders (CFU g^−1^)	1.4 × 10^4^	1.1 × 10^5^

Note: ^a^ TPH = Total Petroleum hydrocarbon, ^b^ WHC = Water holding capacity, ^c^ TOC = Total organic carbon.

**Table 3:** Mean initial and final aliphatic and aromatic fraction concentration changes in soils A and B after 112 days of treatment.

Soil	Hydrocarbons		To	T112 days							
mg kg^−1^	NA		BioS		G + BioS		BioS + BioA	
mg kg^−1^	% loss	mg kg^−1^	% loss	mg kg^−1^	% loss	mg kg^−1^	% loss
Soil A	Aliphatic fractions	>C_8_-C_10_	2.78	0.78	72	0.17	94	0.76	73	0.42	85
>C_10_-C_12_	8.56	4.42	48	0.9	99	6.15	28	2.82	67
>C_12_-C_16_	115	37.9	67	13.9	88	44.7	61	26.7	77
>C_16_-C_35_	11,000	3500	68	1230	89	4090	63	2400	78
>C_35_-C_40_	2720	818	70	904	67	861	68	991	64
**Total**	13,800	4360	68	2150	84	5000	64	3420	75
Aromatic fractions	>C_8_-C_10_	0.27	ND	100	ND	–	ND	–	ND	–
>C_10_-C_12_	1.54	0.41	73	0.34	78	0.35	77	0.22	86
>C_12_-C_16_	15.5	1.9	88	1.49	90	3.29	79	1.15	93
>C_16_-C_21_	10.2	1.5	85	0.64	94	1.68	84	0.42	96
>C_21_-C_35_	ND*	ND	ND	ND	ND	ND	ND	ND	ND
**Total**	27.5	3.81	86	2.47	91	5.32	81	1.82	93
Soil B	Aliphatic fractions	>C_8_-C_10_	2.34	0.23	90	0.14	94	–	–	0.1	96
>C_10_-C_12_	20.5	4.4	79	1.76	91	–	–	1.2	94
>C_12_-C_16_	198	62.7	68	14.1	93	–	–	11.2	94
>C_16_-C_35_	4840	1440	70	300	94	–	–	214	96
>C_35_-C_40_	1010	560	45	192	81	–	–	121	88
**Total**	6070	2070	66	508	92	–	–	348	94
Aromatic fraction	>C_8_- C_10_	29.6	7.02	76	5.66	81	–	–	4.82	84
>C_10_-C_12_	24.9	6.2	75	5.36	79	–	–	4.88	80
>C_12_-C_16_	1830	177	90	70.3	96	–	–	82.3	96
>C_16_-C_21_	840	183	78	118	86	–	–	112	87
>C_21_-C_35_	43.1	10.3	76	8.45	80	–	–	5.23	88
**Total**	2770	384	86	208	93	–	–	209	92

Note: *ND = Not detected.

**Table 4:** Degradation constant rate of the aliphatic and aromatic fractions.

	Degradation constant rate *k*^a^ (d^−1^)			
Soil A		Soil B	
Aliphatic	Aromatic	Aliphatic	Aromatic
Natural attenuation	−0.0084	−0.0016	−0.0065	−0.0021
BioS (Soil A)	−0.0104	−0.0022	−0.0086	−0.0023
Grinding + BioS	−0.0078	−0.0020	–	–
BioS + BioA	−0.0092	−0.0023	−0.0081	0.023

a *k* was calculated from equation: 1n[TPH] = −*kt* + b.
